# MGMT inhibition regulates radioresponse in GBM, GSC, and melanoma

**DOI:** 10.1038/s41598-024-61240-x

**Published:** 2024-05-29

**Authors:** Hong Shik Yun, Tamalee R. Kramp, Kamalakannan Palanichamy, Philip J. Tofilon, Kevin Camphausen

**Affiliations:** 1 Radiation Oncology Branch, National Cancer Institute, 10 Center Drive, 9000 Rockville Pike, Building 10, Bethesda, MD 20892 USA; 2 Department of Radiation Oncology, The Ohio State University Wexner Medical Center, Arthur G. James Comprehensive Cancer Center and Richard J. Solove Research Institute, Columbus, OH USA

**Keywords:** Cancer, Cell biology, Molecular biology, Oncology

## Abstract

Radiotherapy is the standard treatment for glioblastoma (GBM), but the overall survival rate for radiotherapy treated GBM patients is poor. The use of adjuvant and concomitant temozolomide (TMZ) improves the outcome; however, the effectiveness of this treatment varies according to MGMT levels. Herein, we evaluated whether MGMT expression affected the radioresponse of human GBM, GBM stem-like cells (GSCs), and melanoma. Our results indicated a correlation between *MGMT* promoter methylation status and MGMT expression. MGMT-producing cell lines ACPK1, GBMJ1, A375, and MM415 displayed enhanced radiosensitivity when MGMT was silenced using siRNA or when inhibited by lomeguatrib, whereas the OSU61, NSC11, WM852, and WM266-4 cell lines, which do not normally produce MGMT, displayed reduced radiosensitivity when MGMT was overexpressed. Mechanistically lomeguatrib prolonged radiation-induced *γ*H2AX retention in MGMT-producing cells without specific cell cycle changes, suggesting that lomeguatrib-induced radiosensitization in these cells is due to radiation-induced DNA double-stranded break (DSB) repair inhibition. The DNA-DSB repair inhibition resulted in cell death via mitotic catastrophe in MGMT-producing cells. Overall, our results demonstrate that MGMT expression regulates radioresponse in GBM, GSC, and melanoma, implying a role for MGMT as a target for radiosensitization.

## Introduction

Glioblastoma (GBM) is the most aggressive subgroup of malignant gliomas. The median survival of patients with GBM treated with surgical resection alone is approximately six months^1^, and for those treated with the most aggressive combined treatment modalities, including surgery, chemotherapy, and radiotherapy, increases to approximately 14–17 months^2^. This improvement is partially due to the inclusion of temozolomide (TMZ), an oral alkylating agent, as a concomitant therapy to radiation, acting as a radiation modifier^3,4^. However, the combination effect of radiation and TMZ on GBM treatment varies, depending on the presence or absence of O^6^-methylguanine-DNA methyltransferase (MGMT) expression, which is regulated by *MGMT* promoter methylation status^5,6^, a known potential predictor of GBM treatment response to TMZ and a major determinant of a patient’s prognosis^6^.

MGMT is essential for maintaining DNA integrity^7^ and is a key factor in the mechanism of tumor resistance to alkylating agents^8^. Alkylating agents are highly reactive molecules that bind to DNA, and the DNA site they most frequently methylate is the O^6^ position of guanine, which creates O^6^-methylguanine (O^6^-meG)^9,10^. The O^6^-meG produced under these circumstances has cytotoxic and mutagenic potential^11,12^. MGMT recognizes the O^6^-meG base lesion induced by alkylating agents and transfers the methyl adducts from O^6^-meG to itself, restoring the guanine base; then MGMT is auto-inactivated, ubiquitinated, and degraded^13^. Through this mechanism, MGMT induces resistance to alkylating agents, so whether MGMT is expressed in tumors can affect the therapeutic effects of alkylating agents alone or in combination with radiation^6^. MGMT expression is regulated by the methylation of the CpG island within the *MGMT* promoter rather than gene deletion, mutation, rearrangement, or unstable RNA^14–16^. Thus, methylation-specific PCR (MCP) is frequently used to detect the methylation status within the *MGMT* promoter, stratifying patients’ tumor samples into MGMT methylated and unmethylated^17,18^. For reference, *MGMT*^(−)^ means that there is almost no methylation of the *MGMT* promoter and the presence of MGMT protein expression, but in the case of *MGMT*^(+)^, there is methylation of the *MGMT* promoter and no expression of MGMT protein.

*MGMT* inhibitors control MGMT expression and activity in the tumor. Because O^6^-BG, an MGMT inhibitor, can pass through the blood–brain barrier, it has been used in clinical trials as a sensitizer for alkylating agents to the gliomas^19^. Phase I, II, and III clinical trial results of combination O^6^-BG and TMZ treatment of cancers, such as brain tumors, melanoma, lymphoma, and colon cancer, indicated that this combination was effective in tumor suppression^20–23^. However, it has been reported that O6-BG increases the risk of hydrocephalus, CSF leak, and CSF/brain infection in glioblastoma patients^24^ and that combination of O6-BG with other glioblastoma treatments (radiation or BCNU) may cause additional toxicity^25^. Lomeguatrib (O^6^-BTG), another MGMT inhibitor used in this study, not only efficiently inactivated the in vitro and in vivo MGMT level or activity in various tumors, but also effectively increased tumor sensitivity to TMZ^26–29^. Lomeguatrib was recently reported to be effective as a radiosensitizer in GBM cell lines carrying unmethylated *MGMT* promoters^30^, but robust studies to define the role of MGMT itself or MGMT inhibitors on radioresponse had not been performed.

In this study, we evaluated the effects of in vitro molecular biological regulation of MGMT in GBM, GSC, and melanoma cell lines and found that MGMT expression differentially regulates the radioresponse of these tumors. In addition, radioresponse regulation via MGMT control in GBM, GSCs, and melanoma was associated with persistence of radiation-induced *γ*H2AX foci and radiation-induced cell death by mitotic catastrophe, suggesting that MGMT is associated with repair of radiation-induced DNA double-strand breaks (DSBs) in GBM, GSCs, and melanoma.

## Materials and methods

### Cell lines and treatments

We confirm that all methods were carried out in accordance with relevant guidelines and regulations, approved by the NIH safety committee. Adherent glioblastoma cell lines (GBMs [ACPK1 and OSU61]), glioblastoma stem-like cell lines (GSCs [GBMJ1 and NSC11]), and melanoma cell lines (A375, MM415, WM852, and WM266-4) were used in this study. Primary glioblastoma cell lines ACPK1 and OSU61 used in this study were isolated from glioblastoma patient biopsies and authenticated by a neuropathologist at The Ohio State University. These cells were isolated in accordance with The Ohio State University Intuitional Review Boards for IRB (2009C0065 and 2014C0115), and IBC (2009R0169). The neurosphere-forming GBMJ1 and NSC11^31^ (Dr. Frederick Lang, MD Anderson Cancer Center) cells were maintained in stem cell medium (DMEM/F12), supplemented with B27 (Thermo Fisher Scientific), basic fibroblast growth factor (bFGF), and epidermal growth factor (EGF) (50 ng/ml each, Sigma-Aldrich) at 37℃, 5% CO_2_. For in vitro experiments, GBMJ1 and NSC11 neurospheres were disaggregated into single-cell suspensions using TryplE Express enzyme (Thermo Fisher Scientific) and seeded onto Biocoat™ poly-l-lysine-coated multi-well plates (Corning) or chamber slides (Sigma-Aldrich). A375, MM415, WM852, and WM266-4 cells were obtained from the ATCC and grown in DMEM (Invitrogen) and RPMI 1640 (Invitrogen) with 10% fetal bovine serum and maintained at 37℃, 5% CO_2_. Lomeguatrib (O^6^-BTG) purchased from Selleckchem (Cat. S8056) was reconstituted in DMSO (100 mM) and stored at -20℃. Cells were irradiated as monolayer cultures using an XRad 320 X-ray source (Precision XRay Inc.) at a dose rate of 2.5 Gy/min.

### Genomic DNA extraction and methylation-specific PCR analysis

Genomic DNA was isolated using the Monarch Genomic DNA Purification Kit (New England Biolabs), then used for MGMT promoter methylation-specific (MSP) PCR analysis. The DNA was treated with bisulfite using the EZ DNA Methylation-Gold™ Kit (Zymo Research), according to the manufacturer’s instructions. MSP was performed using a nested, two-step approach. The stage-1 PCR products were diluted 50- to 100-fold, then 5 to 10 μl were subjected to stage-2 PCR, using primers specific to the methylated or unmethylated template. Primer sequences used in the stage-1 amplification of MGMT genes^32^, and primer sequences used to selectively amplify unmethylated or methylated alleles of the MGMT genes in the stage-2 PCR have been previously described^33^. The PCR products were resolved using 4% low melting point agarose gels.

### Reverse transcription PCR and quantitative real-time PCR

Total RNA was isolated using Direct-zol RNA Miniprep Kits (Zymo Research). Reverse transcription PCR (RT-PCR) reactions were performed using the ImProm-II™ Reverse Transcription System (Promega) for 5 min at 25 ℃ for annealing, 60 min at 42 ℃ for extension, and 15 min at 70 ℃ for reverse transcriptase inactivation. MGMT mRNA levels were determined by quantitative real-time PCR (qRT-PCR) using the TaqMan™ Universal Master Mix II, with UNG (Thermo Fisher Scientific) and MGMT Taqman probes (Hs00172470_m1; Thermo Fisher Scientific). The amplification signal of the target gene was normalized to that of GAPDH in the same reaction.

### Transient transfection of small interfering RNA

MGMT-specific small, interfering RNA (siRNA) based on the sequence of human *MGMT* was purchased from Qiagen (FlexiTube siRNA—Hs_MGMT_1): sense5′-CCAGACAGGUGUUAUGGAATT-3′ and antisense 5′-UUCCAUAACACCUGUCUGGTG -3′. MGMT-producing cell lines were transfected with 25 nM si (negative control) and siMGMT in a serum-free medium for overnight incubation using Lipofectamine™ RNAiMAX (Thermo Fisher Scientific) according to the manufacturer’s protocol. Cells were further incubated in the growth medium at 37 ℃ for 48 h. MGMT knockdown of was verified by immunoblot analysis.

### Plasmid construction and transfections

Plasmids were constructed via standard recombinant cloning techniques and all changes were verified by DNA sequencing. Human MGMT cDNA (MGMT [untagged]-human O^6^-methylguanine-DNA methyltransferase_wild type; OriGene [cat # SC322190]) was amplified via PCR and cloned into pcDNA3.1-Flag vectors. The pcDNA3.1-Flag-MGMT vector was generated using primers 5ʹ- TGACAAGCTTATGCTGGGACAGCCCGCGCCCCTAGAA-3ʹ (HindIII) and 5ʹ- TCCGAATTCCTAGTTTCGGCCAGCAGGCGGGGAGCCCGA-3ʹ (EcoRI). *MGMT* was then inserted between the HindIII and EcoRI restriction sites in pEGFP-N1 vectors. Transfection with these plasmids was performed using Lipofectamine™ 3000 (Thermo Fisher Scientific) in accordance with the manufacturer’s guidelines. MGMT overexpression levels were determined by immunoblot analysis and imaging using a Zeiss upright fluorescent microscope.

### Clonogenic assays

ACPK1, A375, and MM415 cells were trypsinized and seeded onto six-well tissue culture plates (50 to 3200 cells per well, depending on the radiation dose). After allowing cells time to attach (16 h), cultures received lomeguatrib for 16 h prior to 1–6 Gy irradiation. The lomeguatrib-containing medium was removed 24 h after irradiation, the cells rinsed, and fresh, drug-free media was added. For GSC analysis, GBMJ1 cells were disaggregated into single-cell suspensions and seeded into poly-l-lysine-coated six-well plates (200 to 6,400 cells per well, depending on the radiation dose). After allowing cells time to attach (16 h), cultures received lomeguatrib (100 µM) for 16 h prior to 1–4 Gy radiation. The lomeguatrib-containing medium was removed 24 h after irradiation, the cells rinsed, and fresh, drug-free media was added. Colonies were stained with 0.1% crystal violet 10 to 12 days after seeding ACPK1, A375, and MM415 cells, or 14 to 21 days after seeding GBMJ1 cells. For OSU61, NSC11, WM852, and WM266-4, clonogenic assays were performed as described above 48 h after transfection with the pEGFP-N1-*MGMT* vector, but they were not treated with lomeguatrib. The number of colonies containing at least 50 cells were counted and the surviving fractions calculated. Data presented are the mean ± SE from at least three independent experiments.

### Flow cytometric analysis of cell cycle

Flow cytometry was used to evaluate the cell cycle phase distribution. The drug treatment protocols used were the same as those described for the clonogenic assays. Following treatment of lomeguatrib for 24 h, cells were harvested at various time points, fixed with 70% ethanol, and stained with propidium iodide (Sigma-Aldrich). DNA content for cell-cycle analysis was determined with a LSR Fortessa™ flow cytometer (BD Biosciences).

### Immunoblot analysis

Whole-cell pellets of all cell types were collected in RIPA lysis buffer (Thermo Fisher Scientific) for protein extraction. Total protein was quantified using a BCA protein assay (Thermo Fisher Scientific). Proteins were separated by SDS-PAGE (Mini-Protean TGX™ Gels [Bio-Rad]), transferred to a nitrocellulose membrane (Bio-Rad), and probed with antibodies targeting MGMT (1:1000; abcam [ab108630]), GFP (1:1000; Roche [11814460001]), and β-actin (1:10,000; Cell Signaling [#3700]). Bands were visualized with IR Dye Secondary Antibodies (LI-COR) and quantified using an Odyssey CLx Image System (LI-COR).

### Immunofluorescent staining for *γ*H2AX foci and mitotic catastrophe detection

Visualization of γH2AX foci and mitotic catastrophe was performed as described previously^34^. ACPK1, A375, and MM415 cells were grown on 18 × 18-mm cover glasses and treated with lomeguatrib (100 µM) before irradiation (4 Gy). GBMJ1 cells were grown on 18 × 18-mm cover glasses coated by poly-l-lysine solution (Sigma-Aldrich) and treated with lomeguatrib (50 µM) for 16 h before irradiation (4 Gy). Cells were imaged using a Zeiss upright fluorescent microscope and γH2AX foci were counted in 50 cells per treatment group using Image J. In the case of mitotic catastrophe, nuclear fragmentation was defined as the presence of ≥ 2 lobes within single cells, and 100 cells were scored for each condition. Data presented are the mean ± SEM for three independent experiments. Statistical significance was calculated by Student’s t-test.

## Results

### *MGMT* promoter methylation status and MGMT expression is a non-binary function in GBMs and Melanoma

**Figure 1:**
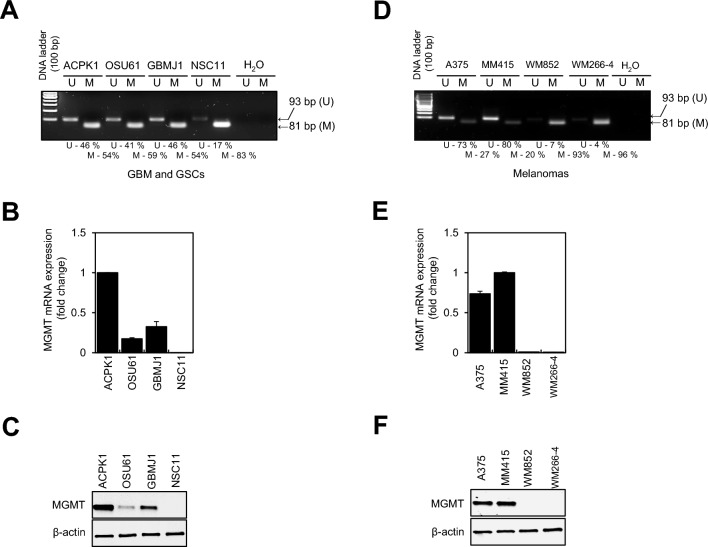
MGMT expression levels in GBM, GSCs and melanoma cell lines. (**A** and **D**) The methylation-specific PCR (MSP) analyses of the *MGMT* promoter from GBMs, GSCs, and melanoma cell lines. Note the bands in the unmethylated (U, 93 bp) and methylated (M, 81 bp) lanes for GBM, GSCs, and melanoma cell lines, reflecting the unmethylated/methylated *MGMT* promoter. Percentages represent the proportion of methylation (M) and unmethylation (U) on the MGMT promoter in each cell line. (**B** and **E)** Transcript levels of MGMT mRNA in GBM, GSCs, and melanoma cell lines were determined by qRT-PCR. (**C** and **F)** MGMT levels in GBM, GSCs, and melanoma cell lines were determined by immunoblot analysis. Data are the mean ± SEM for three independent experiments.

MGMT promoter methylation status^35,36^ detected using methylation-specific PCR (MSP) shown in Fig. 1A exhibits mixed methylated/unmethylated status for three *MGMT*^(−)^ GBMs—ACPK1, OSU61, and GBMJ1 cells. The NSC11 *MGMT*^(+)^ promoter was almost exclusively methylated. Likewise, the two *MGMT*^(−)^ melanoma cell lines, A375 and MM415, showed mixed methylated/unmethylated results for their promoters. The *MGMT*^(+)^ melanoma cell lines, WM852 and WM266-4, showed mostly methylated promoters (Fig. 1D). To determine whether the degree of methylation of the *MGMT* promoter affects the MGMT at translational and transcriptional levels, we performed qRT-PCR and immunoblot analysis on each cell line. As expected, the relative amount of mRNA was proportional to the unmethylated promoter band for both histologies (Fig. 1B and E). Likewise, the amount of protein measured was proportional to the level of mRNA (Fig. 1C and F). These results suggest that *MGMT* promoter methylation is not a binary function and that MGMT protein level gradations exist within *MGMT*^(−)^ cells.

### Silencing MGMT enhances radiosensitivity in GBM and melanoma cells expressing MGMT

**Figure 2:**
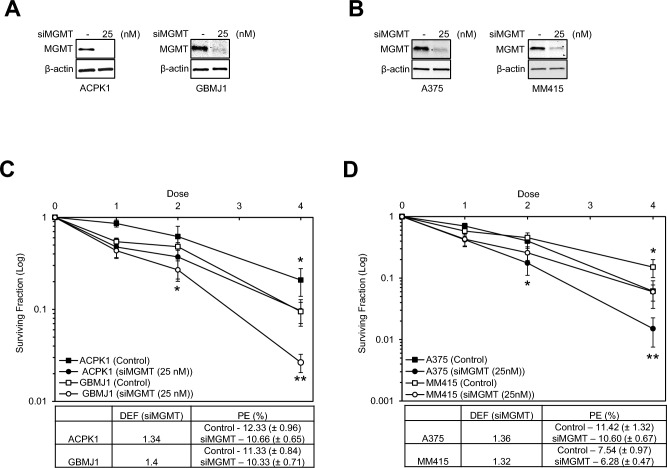
The effect of siMGMT on the radioresponse of MGMT-producing cells. (**A** and **B**) ACPK1, GBMJ1, A375, and MM415 cells were transfected with or without 25 nM siMGMT for 48 h. MGMT levels were determined by immunoblot analysis. (**C** and **D**) ACPK1, GBMJ1, A375, and MM415 cells were transfected with 25 nM of si (negative control) and siMGMT before radiation. Surviving fraction (Log) curves were generated after normalizing for the cytotoxicity generated by siMGMT alone. Data are the mean ± SEM for three independent experiments. * P < 0.05 and ** P < 0.005 by Student’s t-test.

To determine whether MGMT expression is related to radioresponse, clonogenic cell survival assays were performed using MGMT-producing cells (ACPK1, GBMJ1, A375, and MM415). MGMT-producing cells were transfected with 25 nM si (negative control) and siMGMT for 48 h before performing clonogenic cell survival assays. Immunoblot analysis showed that siMGMT downregulated MGMT expression in MGMT-producing cells (Figs. 2A and B). Clonogenic cell survival assays of MGMT-producing cells after siMGMT transfection showed an increase in the radiosensitivity of ACPK1, GBMJ1, A375, and MM415 cells, with dose-enhancement factors (DEFs) ranging from 1.32 to 1.4 (Figs. 2C and D). The siRNA concentration we selected was confirmed to have no additional cytotoxicity as measured by a decrease in plating efficiency (PE) of each cell line. Additionally, to exclude off-target effects due to siMGMT, we transfected siMGMT into NSC11, which are non-MGMT-producing cells, and then performed a clonogenic assay, and as a result, we confirmed that siMGMT had no effect on the radioresponse of NSC11 cells (Supplmentary Fig. S3). These data indicate that the depletion of endogenous MGMT by siMGMT increases the radiosensitivity of MGMT-producing cells after irradiation.

### Lomeguatrib inhibits MGMT and enhances the radiosensitivity of MGMT-producing cell lines

**Figure 3:**
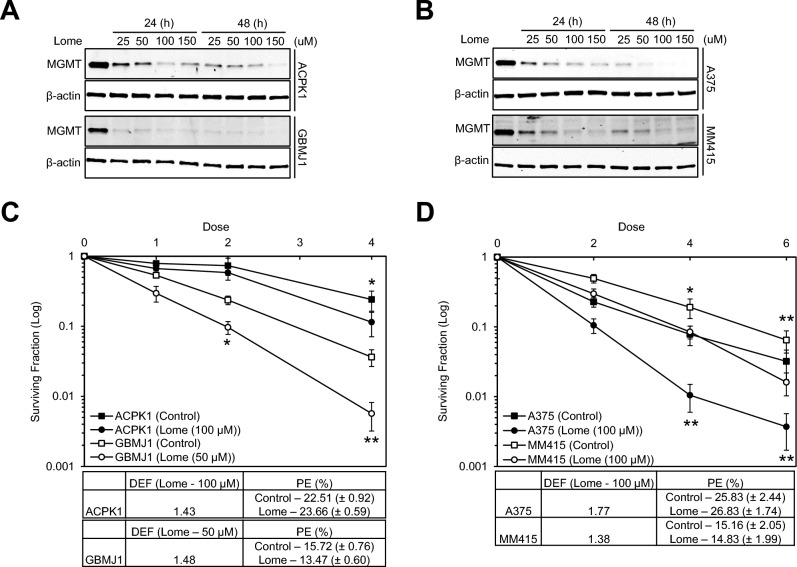
The effect of lomeguatrib on radioresponse of MGMT-producing cells. (**A** and **B**) ACPK1, GBMJ1, A375, and MM415 cells were treated with a gradient (25 µM, 50 µM, 100 µM, and 150 µM) of lomeguatrib for the indicated times (24 h and 48 h). MGMT levels were determined by immunoblot analysis. (**C** and **D**) ACPK1, GBMJ1, A375, and MM415 cells were treated with the designated concentrations of lomeguatrib (ACPK1, A375, and MM415—100 µM and GBMJ1—50 µM) for 16 h before radiation. Surviving fraction (Log) curves were generated after normalizing for the cytotoxicity generated by lomeguatrib alone. * P < 0.05 and ** P < 0.005 by Student’s t-test.

Clonogenic survival assays were conducted to determine whether lomeguatrib, an MGMT inhibitor that promotes MGMT degradation through the ubiquitin–proteasome pathway^37^, increases the radiosensitivity of MGMT-producing cells. MGMT immunoblot analysis was used to determine the lomeguatrib concentration and exposure time required to inhibit MGMT Fig. 3A and B show MGMT-producing cells that were treated with various concentrations of lomeguatrib (25, 50, 100, and 150 µM) for 24 or 48 h, and the results showed a significant reduction in MGMT expression levels 24 h after lomeguatrib treatment. A dose- and time-dependent decrease in MGMT levels was observed at all tested concentrations. Based on these results, clonogenic survival assays of lomeguatrib-treated cells were performed, and, as shown in Figs. 3C and D, the results indicated that treatment with lomeguatrib increased the radiosensitivity of ACPK1, GBMJ1, A375, and MM415 cells, with DEFs at a surviving fraction of 0.1 ranging from 1.38 to 1.77. The lomeguatrib concentration we selected was confirmed to have no cytotoxicity when measured by plating efficiency (PE) of each cell line. These data suggest that inhibition of MGMT using lomeguatrib increased radiosensitivity, recapitulating the siMGMT studies.

### Lomeguatrib increases radiation-induced DNA damage and mitotic catastrophe without affecting the cell cycle in MGMT-producing cell lines

**Figure 4:**
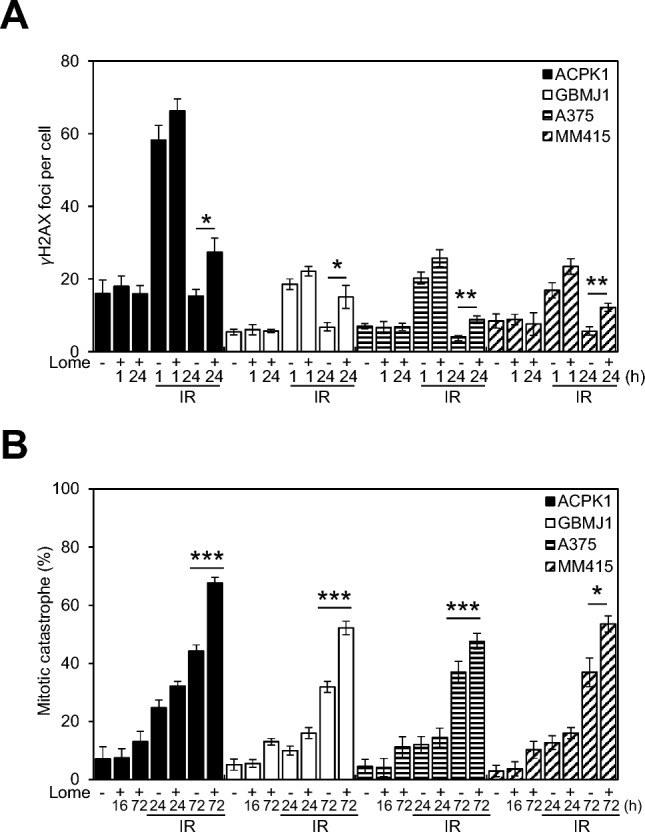
The effect of lomeguatrib on radiation-induced γH2AX foci and mitotic catastrophe in MGMT-producing cells. (**A**) The quantitative assessment of γH2AX foci per cell at 1 h and 24 h after radiation is shown. Foci were counted in at least 50 cells per experiment and representative histograph images obtained from control, lomeguatrib-only, radiation (4 Gy)-only, and lomeguatrib/radiation combination treatment. Data are the mean ± SEM for three independent experiments, and statistical significance was determined by Student’s t-test. *P < 0.05 and **P < 0.005 (radiation versus lomeguatrib/radiation). (**B**) The quantitative assessment of nuclear fragmentation per cell at 24 h and 72 h after radiation is shown. Nuclear fragmentation (defined as the presence of two or more distinct lobes within a single cell) was evaluated in at least 100 cells per treatment per experiment and representative histograph images obtained from control cells and cells pretreated with lomeguatrib alone, radiation (4 Gy) alone, and combination treatment of lomeguatrib with radiation. Data are the mean ± SEM for three to four independent experiments. Statistical significance was determined by Student’s t-test. *P < 0.05 and ***P < 0.0005 (radiation versus Lomeguatrib + radiation).

To determine lomeguatrib’s mechanism of radiosensitization of MGMT-producing cells, the presence of γH2AX foci, an indicator of DNA damage^38^, and its dispersal, which is associated with radiation-induced DNA damage repair^39^, were evaluated. Cells were treated with lomeguatrib (ACPK1, A375, and MM415 at 100 µmol/L and GBMJ1 at 50 µmol/L) prior to radiation (4 Gy), and γH2AX foci determined at 1 and 24 h post-radiation. As shown in Fig. 4A and supplementary Fig. S2A, when MGMT-producing cells were pretreated with lomeguatrib alone, there was no significant increase in the number of γH2AX foci, indicating that drug treatment alone did not cause DNA damage. Radiation treatment led to a significant increase in the number of γH2AX foci at 1 h, which decreased to background levels by 24 h as the DNA damage was repaired. In each cell line, combined radiation and lomeguatrib treatment resulted in a significantly higher number of foci at 24 h compared to cells receiving only radiation, which is consistent with DNA damage repair inhibition and radiosensitization (Fig. 4A and Supplementary Fig. S4A).

Cell cycle distribution was measured to determine whether the lomeguatrib-induced radiosensitization was due to a redistribution of cells into a more sensitive phase of the cell cycle. As shown in Supplementary Fig. S2, there was essentially no consistent difference in the cell cycle distribution with lomeguatrib treatment alone compared to the control (DMSO) for each cell line. Figure 4A and Supplementary Fig. S2 and S4A suggest that combination treatment with radiation and lomeguatrib can attenuate the cellular repair of radiation-induced DNA damage without redistributing into a more sensitive phase of the cell cycle.

To investigate whether lomeguatrib affected radiation-induced mitotic catastrophe, a common death mechanism in cells with unrepaired DNA-DSBs, immunostaining using α-tubulin was performed. Figure 4B and Supplementary Fig. S4B show that, at 16 or 72 h, the percentage of all cells undergoing mitotic catastrophe after treatment with lomeguatrib alone was insignificant compared to the control. However, there was an increase in the number of lomeguatrib and radiation-treated cells undergoing mitotic catastrophe at 72 h in all cells compared to the irradiation-only cells. Therefore, our data, in combination with previous γH2AX results, suggest that the attenuation of radiation-induced DNA damage repair by lomeguatrib leads to mitotic catastrophe.

### Intracellular overexpression of MGMT increases the radioresistance of non-MGMT-producing cells

**Figure 5:**
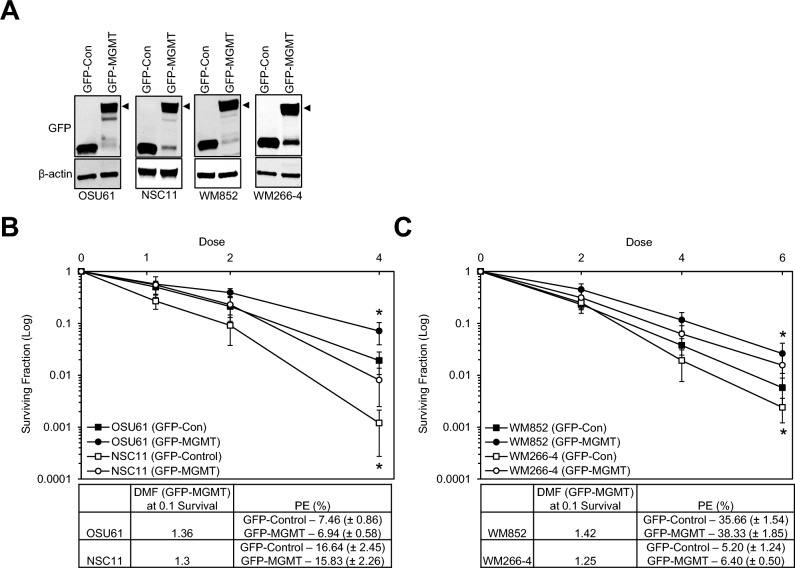
The effect of MGMT overexpression on the radioresponse in non-MGMT-producing cells. (**A**) MGMT protein expression was assessed via western blot analysis following transfection of 1 µg GFP-MGMT vector in non-MGMT-producing cells (OSU61, NSC11, WM852 and WM266-4) for 48 h. (**B** and **C**) OSU61, NSC11, WM852 and WM266-4 cells were transfected with 1 µg of GFP-control vector and GFP-MGMT vector before radiation. Surviving fraction (Log) curves were generated after normalizing for the cytotoxicity generated by GFP-*MGMT* vector alone. DMF values were calculated at a surviving fraction (Log) of 0.1. Data are the mean ± SEM for three independent experiments. * P < 0.05 by Student’s t-test.

To further define the role of MGMT on radioresponse, MGMT was exogenously added to non-MGMT-producing cells (GBM [OSU61], GSCs [NSC11] and melanoma cells [WM852 and WM266-4]) and assessed using a clonogenic cell survival assay. As shown in Fig. 5A and Supplementary Fig. S5, non-MGMT-producing cells were transfected with 1 µg GFP-MGMT (WT) vector for 48 h, and the immunoblot analysis/histograph results showed that exogenous GFP-MGMT (WT) expression was predominantly localized to the nucleus. As shown in Figs. 5 B and C, transfection with the GFP-*MGMT* (WT) vector increased the radioresistance of OSU61, NSC11, WM852, and WM266-4 cells with DMFs at a surviving fraction of 0.1, ranging from 1.25 to 1.42. Therefore, our data indicates that overexpression of exogenous MGMT in non-MGMT-producing cells increases the radioresistance of these cells.

## Discussion

Radiotherapy in combination with the alkylating agent temozolomide (TMZ) has been the standard treatment for most patients with glioblastoma (GBM) for almost 20 years^5^. This combination therapy led to a statistically significant improvement in overall survival; however, the absolute effect was only 2.5 m. In a complementary biomarker paper, it was shown that the methylation status was correlated to RT/ TMZ response rate, as patients with a methylated *MGMT* promotor had a survival benefit compared to those with unmethylated *MGMT* promotors^6^. Additionally, it was shown that the methylation status of the *MGMT* promoter also predicted for survival with irradiation (RT) alone. Of course, because there is no treatment group that did not receive radiation, factors other than the methylation of the MGMT promoter can not be ruled out. However, patients with a methylated *MGMT* promoter who only received RT survived longer than those patients with unmethylated tumors (15.3 m vs. 11.8 m). Thus, MGMT may play a role in the standard response to irradiation and evaluation of this served as the basis for our study.

Through MSP, qRT-PCR, and immunoblot analysis, we confirmed that there was a correlation between the methylation status of the *MGMT* promoters in each of our cell lines—GBM (ACPK1 and OSU61), GSCs (GBMJ1 and NSC11), and melanoma (A375, MM415, WM852, and WM266-4)—and their MGMT mRNA and protein expression (Fig. 1). Although there have been reports of mismatches between the methylation status of the *MGMT* promoter and endogenous MGMT expression^40^, the methylation status of the *MGMT* promoter dictates the use of TMZ in brain tumor care. However, our data also showed that the presence of MGMT protein was not binary but showed a variable level within *MGMT*^(−)^ cell lines. Thus, our cell lines were classified as MGMT-producing (GBM [ACPK1], GSCs [GBMJ1], and melanoma [A375 and MM415]) or non-MGMT-producing (GBM [OSU61], GSCs [NSC11, and melanoma [WM852 and WM266-4]). To evaluate whether in vitro depletion of MGMT in human GBM, GSCs, and melanoma regulated their radioresponse, clonogenic survival assays were performed and showed that the radiosensitivity of each *MGMT*^(−)^ cell line was significantly different when MGMT was depleted or inhibited. Moreover, we showed an enhancement of radioresistance when exogenous MGMT expression in non-MGMT-producing cells is induced via introducing a GFP-MGMT vector. Together, these results suggest that MGMT protein plays a role in the radioresponse of both glioma and melanoma cell lines.

To translate our findings to the clinic, we used the MGMT inhibitor lomeguatrib. Lomeguatrib treatment of MGMT-producing cell lines did not significantly affect the distribution of cells within the cell cycle and, thus, did not cause a redistribution of cells into a more sensitive phase of the cell cycle. This is consistent with previous work, which showed that lomeguatrib treatment of GBM cell lines and human anaplastic astrocytoma cell lines does not induce specific phase changes in the cell cycle^41,42^. Next, we evaluated DNA DSB repair by measuring γH2AX foci. γH2AX foci form at DNA DSBs and can be used as a sensitive indicator of DSB formation and repair^39,43–45^. Our study shows the number of γH2AX foci in the MGMT-producing cell line of the lomeguatrib-only treatment group was not different from that of the control group, that there was no difference in the number of γH2AX foci between the radiation-only and combination treatment groups after 1 h of radiation, and that the number of γH2AX foci in the lomeguatrib/radiation combination treatment group of MGMT-producing cell lines was significantly greater at 24 h after radiation than that of individual treatments. These data suggest that lomeguatrib treatment alone does not cause DNA DSB, the combination treatment does not cause more breaks than RT alone, but that the lomeguatrib treatment leads to an inhibition of DSB repair. Additionally, as shown here, lomeguatrib treatment of MGMT-producing cell lines significantly increased radiation-induced mitotic catastrophe. Therefore, we suggest that the cause of the significant increase in radiation-induced radiosensitivity by lomeguatrib treatment of MGMT-producing cell lines is due to non-repaired DNA DSBs leading to cellular death through mitotic catastrophe.

### Supplementary Information

Supplementary Information.

## Data Availability

All data generated and analyzed in this study are included in the manuscript and its supplementary files.
