# Supplementary material for: MGMT inhibition regulates radioresponse in GBM, GSC, and melanoma
Source: Sci Rep. 2024 May 29;14:12363. doi: 10.1038/s41598-024-61240-x (PMC11136993; doi:10.1038/s41598-024-61240-x)
Supplement: Supplementary file 1 — Supplementary Information. [file 41598_2024_61240_MOESM1_ESM.docx]

**Supplementary Figures S1**


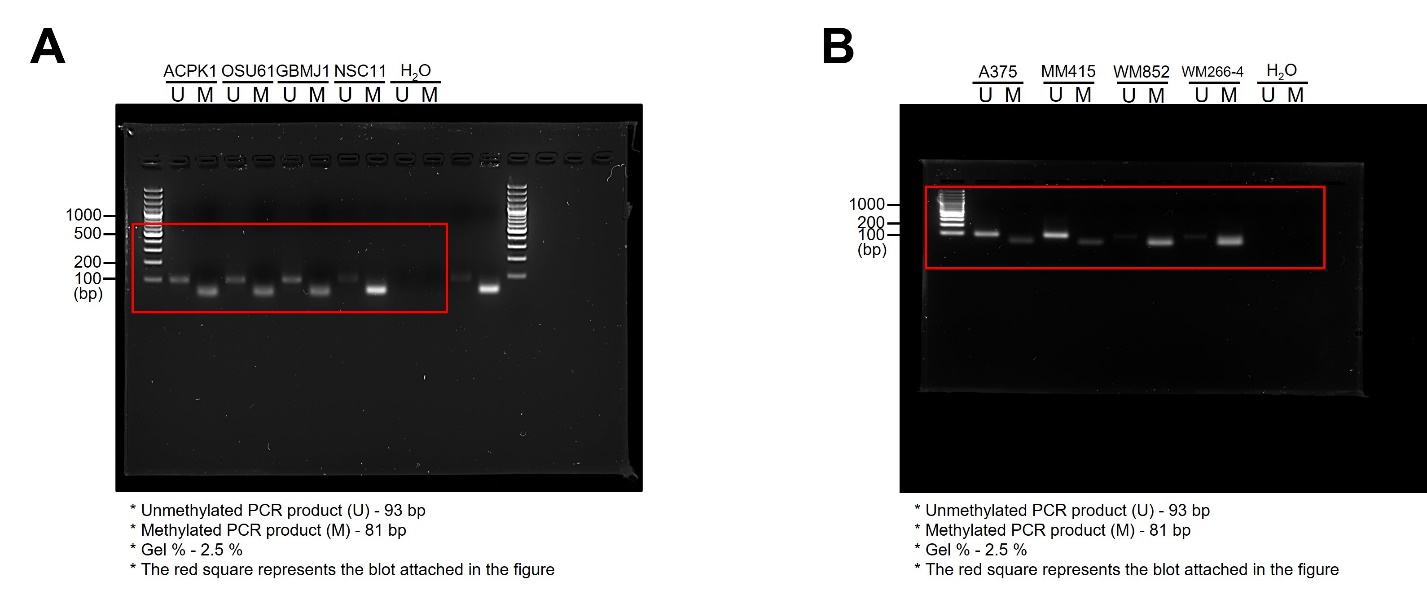


**
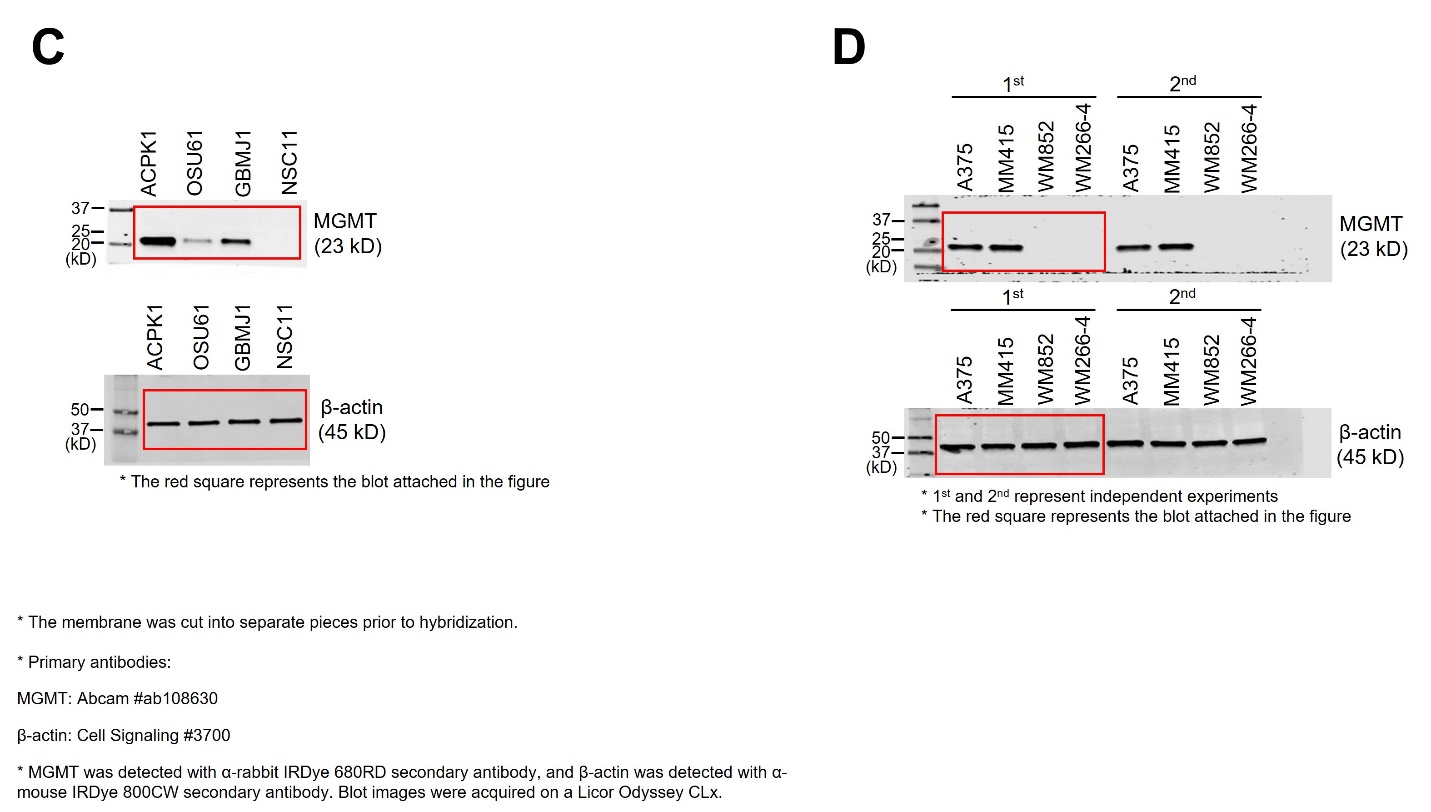
**

**
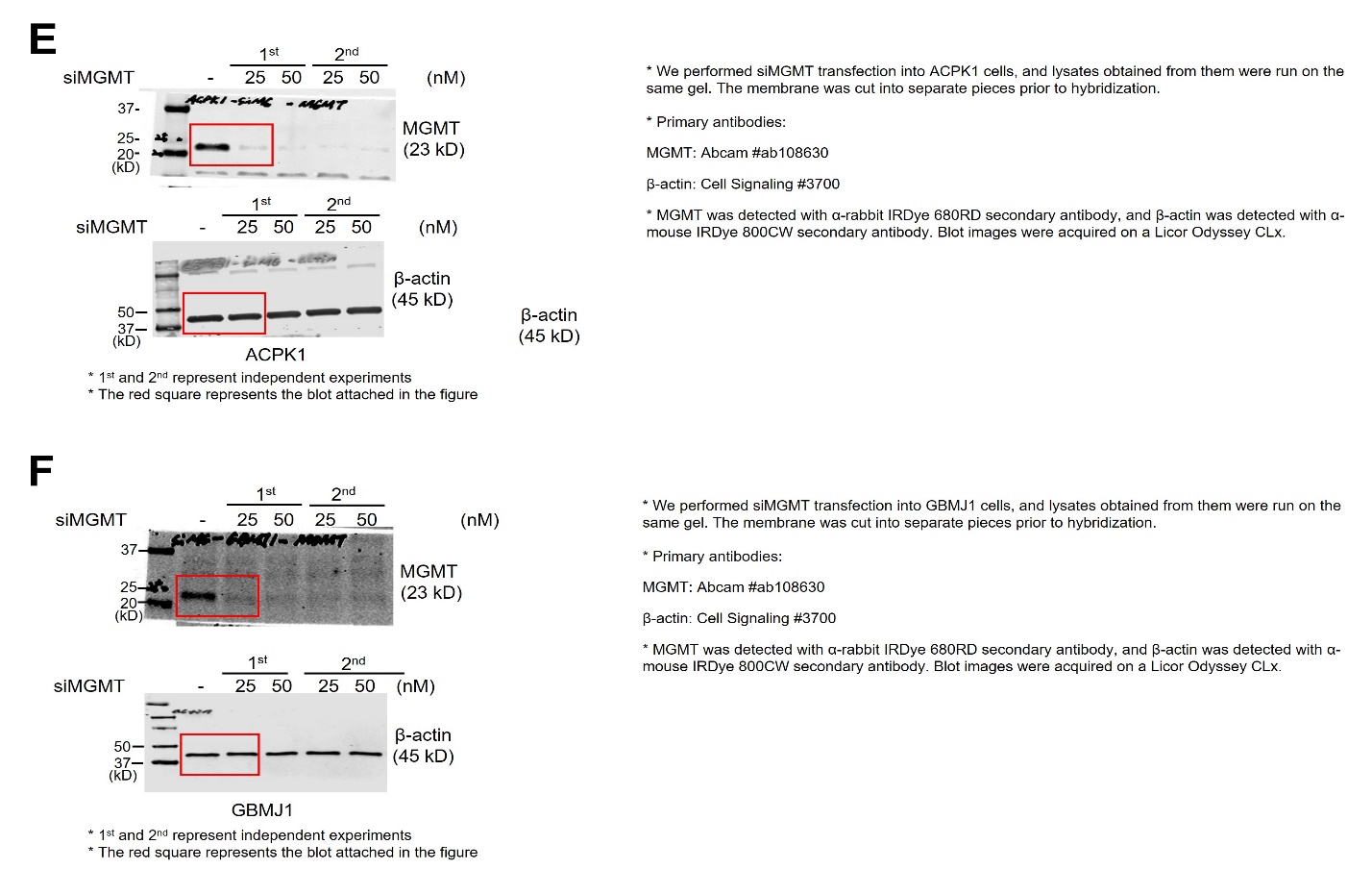
**

**
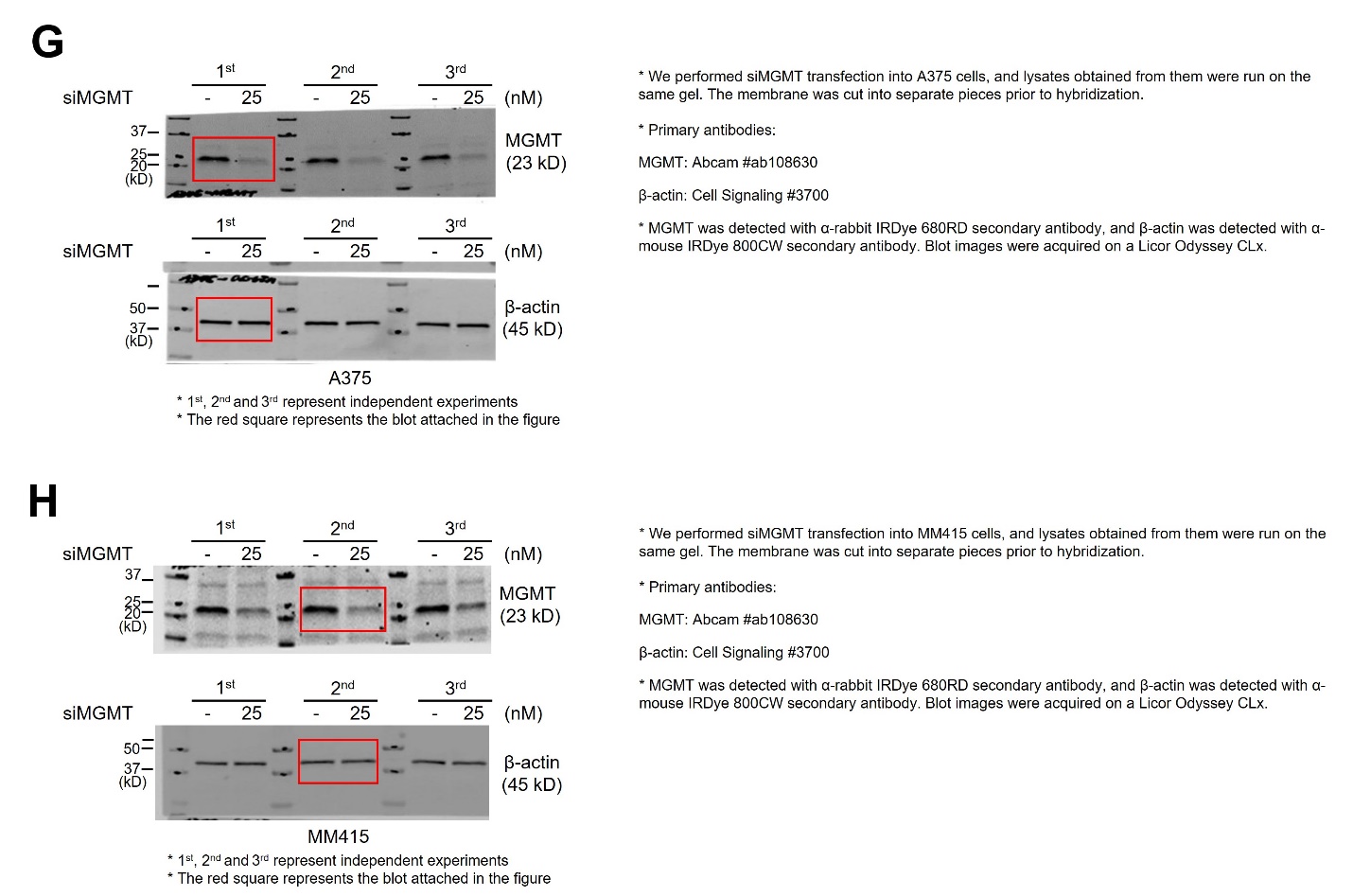
**

**
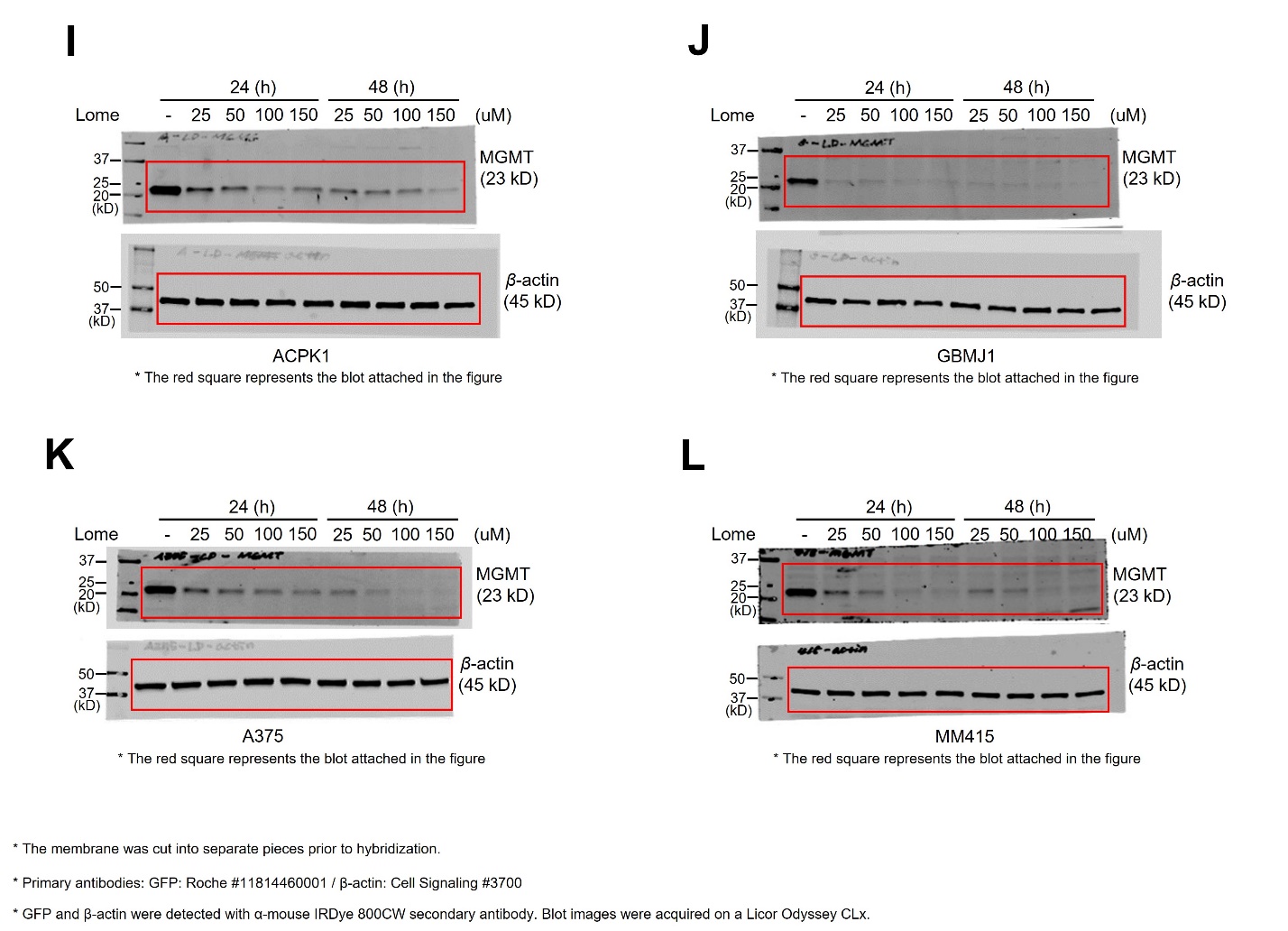
**

**
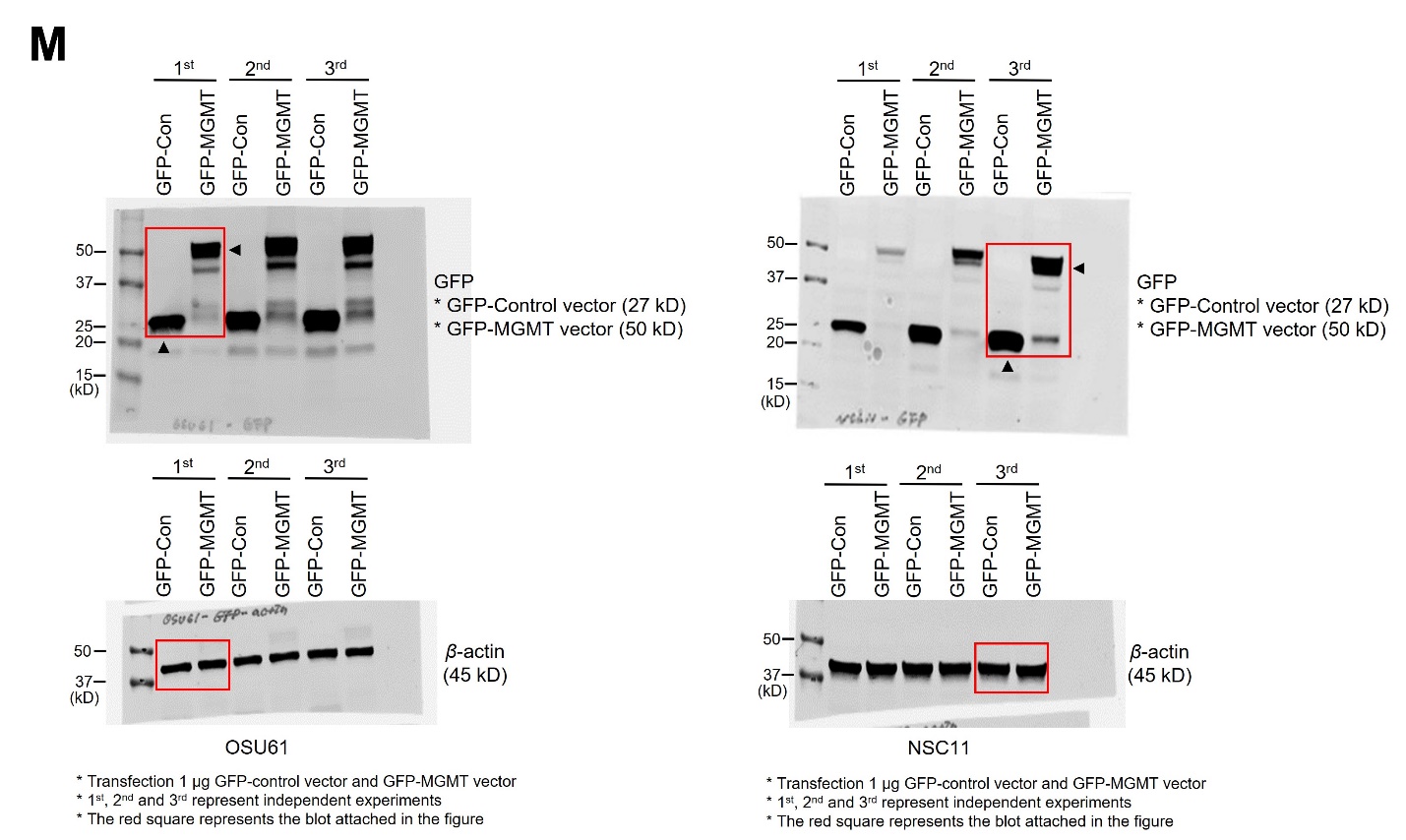
**

**
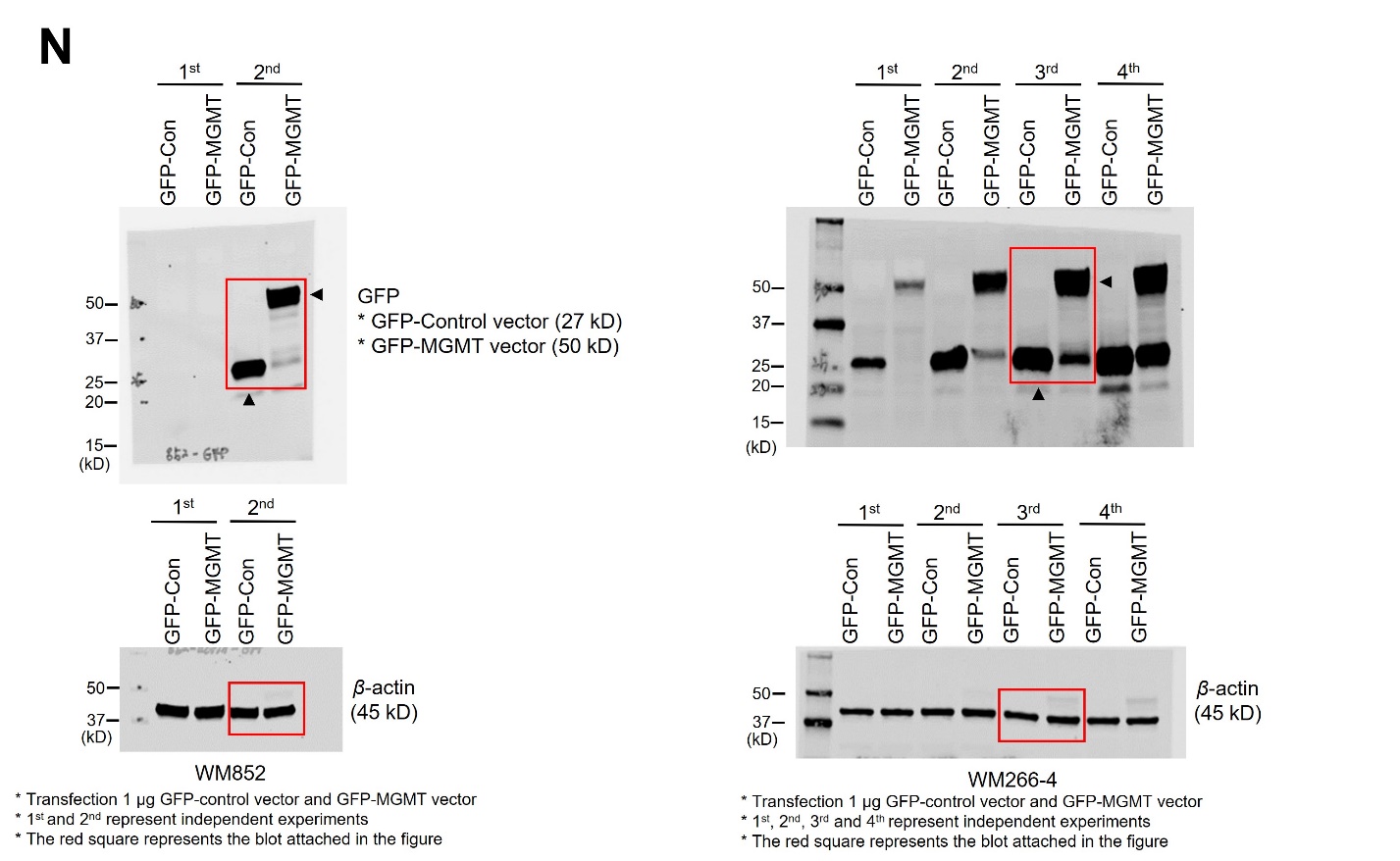
**

**Supplementary Figure S1.** We present original images of gels and immunoblots in all figures. (A) Full gel for figure 1A. (B) Full gel for figure 1D. (C) Full immunoblot for figure 1C. (D) Full immunoblot for figure 1F. (E-F) Full immunoblot for figure 2A. (G-H) Full immunoblot for figure 2B. (I-J) Full immunoblot for figure 3A. (K-L) Full immunoblot for figure 3B. (M-N) Full immunoblot for figure 5A. Depending on the experimental situation (molecular weight of target proteins or number of loading samples, etc.), the blot was cut before hybridization with antibodies.

**Supplementary Figures S2**

**Supplementary Figure S2.** The effect of lomeguatrib on cell cycle in MGMT-producing cells. The percentage of cells in G0/G1, S, and G2/M-phase of the cell cycle by time point after treatment with lomeguatrib (ACPK1, A375, and MM415–100 µM and GBMJ1–50 µM). Data are the mean ± SEM for three independent experiments.

**Supplementary Figures S3**


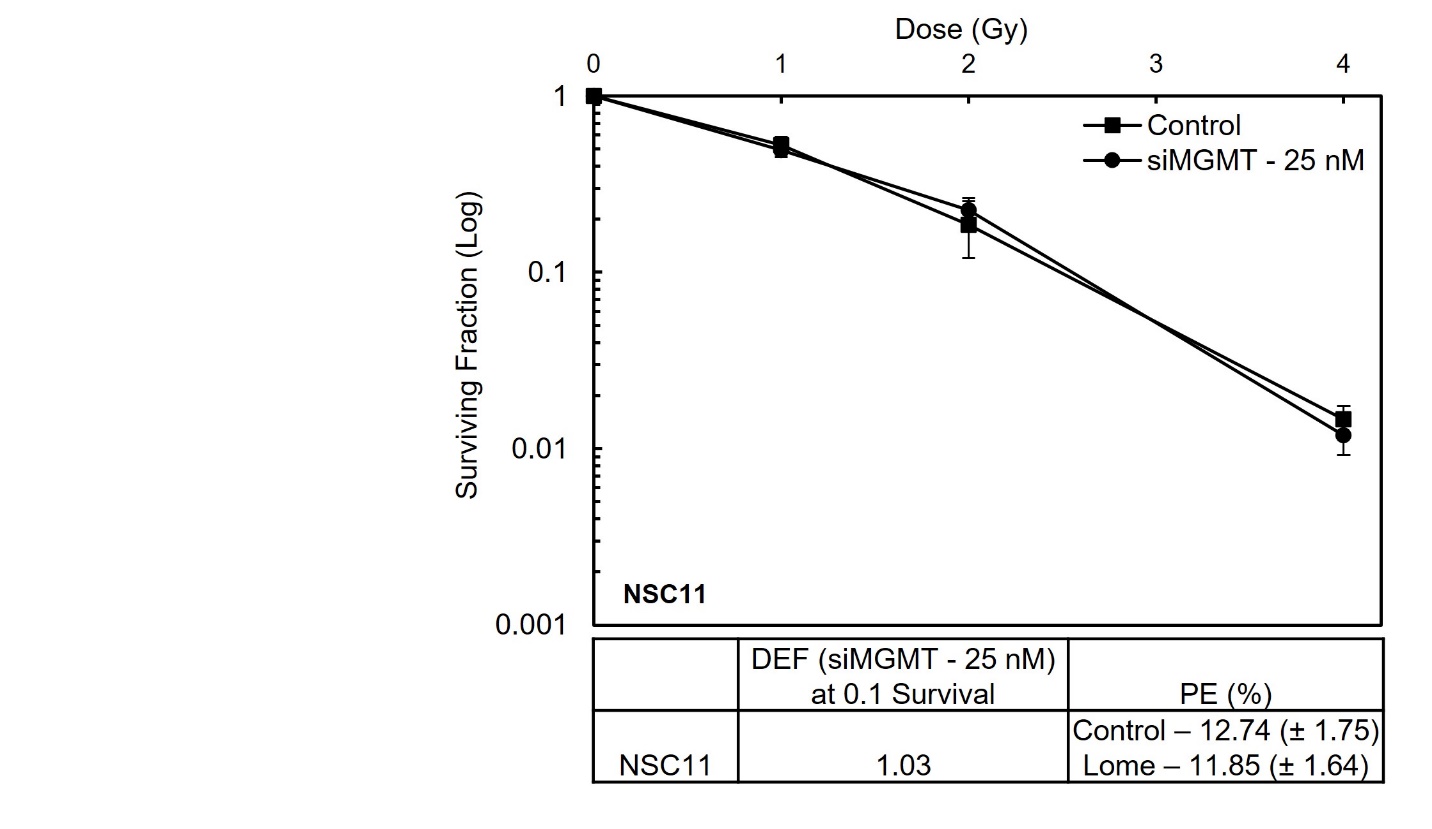


**Supplementary Figure S3.** The effect of siMGMT on the radioresponse of non-MGMT-producing cells. NSC11 cells were transfected with or without 25 nM siMGMT for 48 h. NSC11 cells were transfected with 25 nM of si (negative control) and siMGMT before radiation. Surviving fraction (Log) curves were generated after normalizing for the cytotoxicity generated by si treatment alone. Data are the mean ± SEM for three independent experiments.

**Supplementary Figures S4**


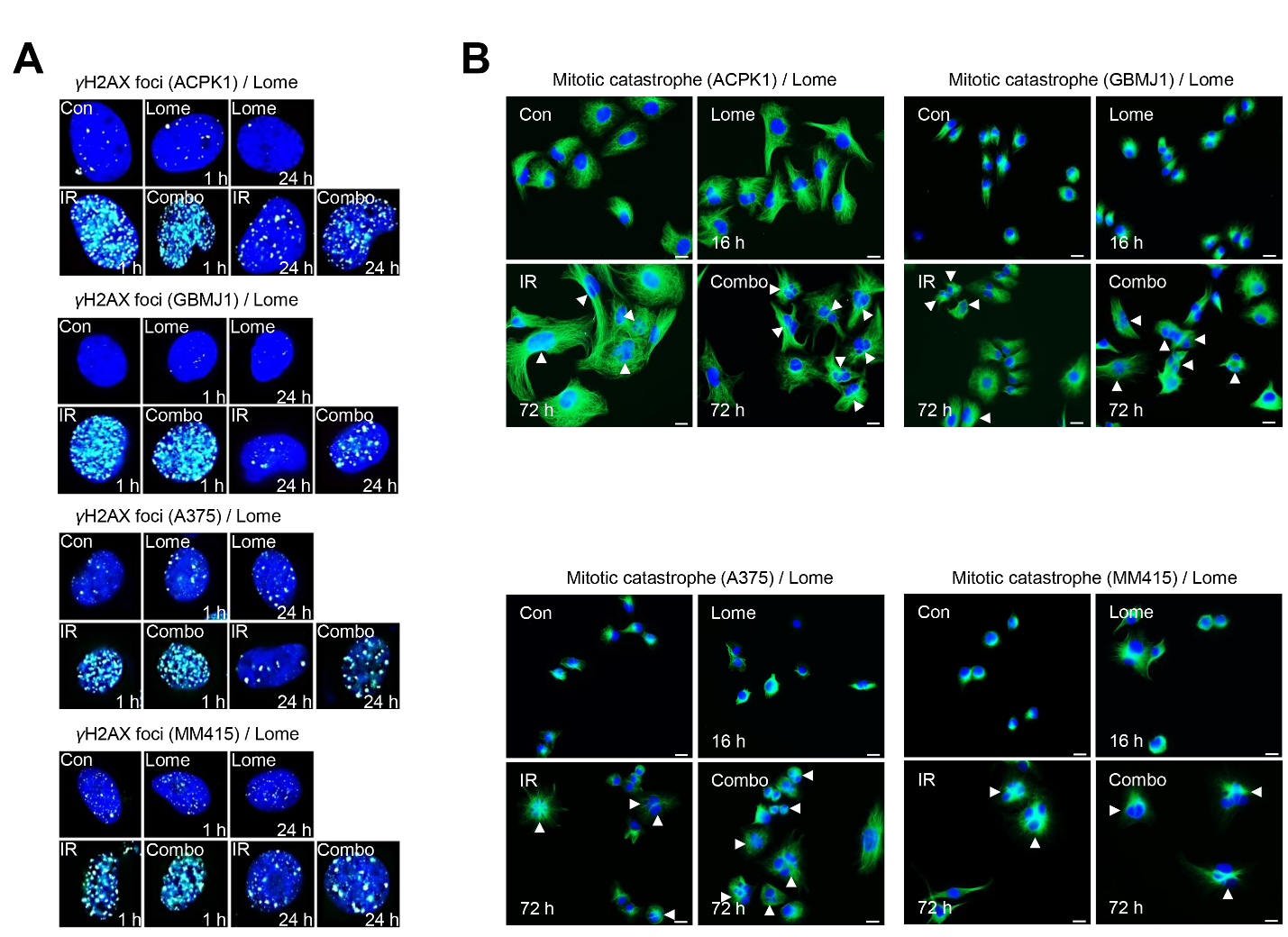


**Supplementary Figure S4.** The histograph of radiation-induced γH2AX foci and mitotic catastrophe in MGMT-producing cells. (A) Representative γH2AX foci histograph images obtained from control cells and cells treated with lomeguatrib (ACPK1, A375, and MM415 – 100 µM and GBMJ1 – 50 µM); 1 h and 24 h) alone, Radiation (4 Gy; 1 h and 24 h) alone, and lomeguatrib (ACPK1, A375, and MM415 – 100 µM and GBMJ1 – 50 µM); 1 h and 24 h before radiation) plus radiation at 1 h and 24 h after 4 Gy radiation. (B) Representative mitotic catastrophe histograph images obtained from control cells and cells treated with lomeguatrib (100 µM (GBMJ1–50 µM); 16 h) alone, radiation (4 Gy; 72 h) alone, and lomeguatrib (ACPK1, A375, and MM415 – 100 µM and GBMJ1 – 50 µM); 16 h before radiation) plus radiation at72 h after 4 Gy radiation. Scale bar = 20 μm

**Supplementary Figures S5**


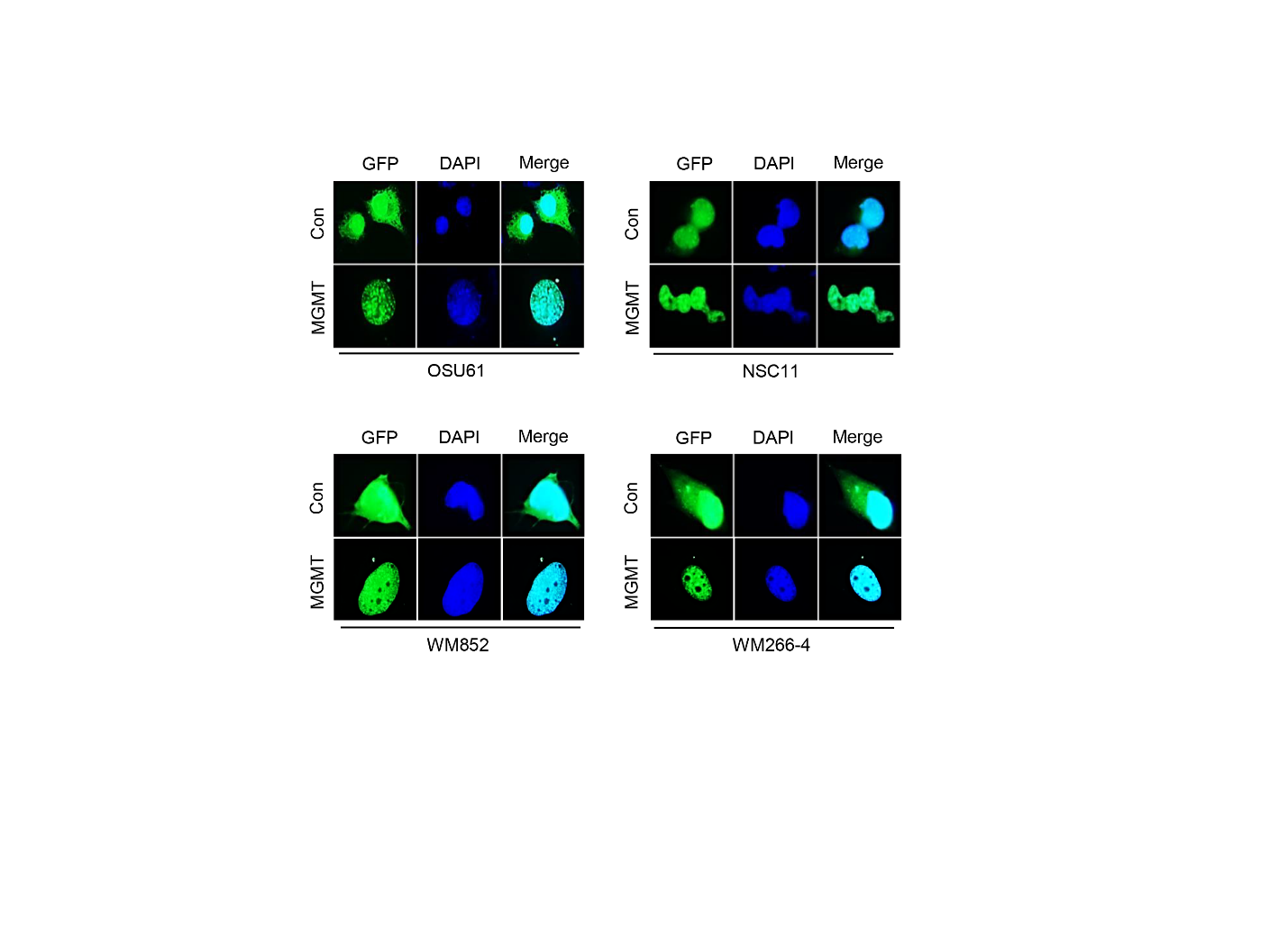


**Supplementary Figure S5.** The histography of GFP-*MGMT* expression vector in non-MGMT-producing cells. Non-MGMT-producing cells (OSU61, NSC11, WM852 and WM266-4) were transfected with GFP-control and GFP-*MGMT* vectors for 48 h. Localization of GFP-control and GFP-*MGMT* was visualized using a Zeiss upright fluorescent microscope, and representative images are shown.
